# Treatment of Periostitis and Alveolar Abscess

**Published:** 1859-06

**Authors:** E. Collins


### TREATMENT OF PERIOSTITIS AND ALVEOLAR ABSCESS.

BY E. COLLINS.

The remedial agents which I have found by experience to be the most efficacious in overcoming the above named morbid conditions, are principally of a surgical nature. Yet, while I view means of a surgical nature of primary importance to all other exclusive remedies, because by such means we get at once at the cause, which is as important, if we hope to accomplish a speedy and permanent cure of the diseases mentioned above, as it is in the treatment of any other diseases, there are remedies which operate indirectly, that may be often times advantageously employed in conjunction with the surgical remedies, or may, in some cases where they are not employed in conjunction with the latter, prove effectual in counteracting periotitis in its forming stage.

But every experienced dentist is fully aware that it matters not how well directed his skill, or how well adapted the remedies employed, to meet every feature in the case under treatment, he is often liable to fail in accomplishing the desired results, owing to the following several conditions, which, if carefully observed, serve us as a basis for pretty correct prognostication, so that we may know what to promise our patient, and whether to adopt the grand specific of extraction, and thus relieve him of unnecessary suffering, at the sacrifice of the offending organ, or whether to institute such treatment as may result in its salvation:

First, the location of the organ affected; 2d, the constitution of the patient; 3d, the patient's habits as respects the use of intoxicating beverages; 4th, the patience and resolution exercised by the sufferer in submitting to treatment.

Taking the several conditions into consideration, we find the inferior and superior molars more difficult of successful treatment than the teeth anterior to them, in consequence of the greater difficulty in reaching the several points at which those teeth are liable to become affected, and that, too, often at the same time.

Again, the superior teeth can be more successfully treated, especially where suppuration has begun and proceeded to any considerable extent, than can the inferior teeth; because of the more ready and thorough exit of the accumulated matter, through the opening made for its escape.

Again, those of low recuperative powers, which is a condition consequent upon a depressed or impoverished state of the blood, principally induced either by evil hereditary influences, or vicious practices, such as scrofula, and habitual inebriation, too frequent, and oft repeated impure sexual intercourse; with such we need not expect to be as successful as with those whose blood is rich and copious, and who abstain from such base life and health destroying and soul damning vices, as the two last mentioned.

Again, we can not expect as much success in those cases where the patient will not submit to thorough treatment, and yield strict obedience to what is required of him, as with one who behaves differently. This last is of no trivial importance, and any rational or philosophic mind will at once perceive that it is just as important that a sore and aching tooth should be as patiently and carefully nursed by the sufferer, as any other diseased member of the body.

The readiness with which the difficulty can be removed by extraction, and the want of a proper appreciation of the teeth, lead the patient often to promptly demand of the dentist the performance of this operation, even when the location of the organ, the constitution of the patient, his habits, etc., all combine in affording reasons to hope for success under a due course of treatment. This obstinacy and ignorance on the part of patients, is a great impediment in the way of our rapid advancement in the healing department of our profession, and can only be overcome by the general diffusion of knowledge in regard to the teeth among the people.

Having briefly described the conditions upon which we are to base our prognostications for success or failure, I here present in brief, my practice in the treatment of the diseases under consideration. First, I ascertain the location of the apex of the root affected, which may be pretty accurately done by pressure over the gum with the point of the finger. If there be the least tumefaction of the soft tissues external to the alveolus, it is thus readily detected. Should there be no external enlargement, then we have to rely upon the sensibilities of the patient, who will signify to us when we touch the most sensitive point, which is always opposite the seat of the difficulty, or apex of the fang affected. Having obtained this information, the lancet is thrust in at this point, cutting to the bone, and drawing it toward the crown of the tooth, making an incision about a half an inch in length; then, with a blunt, but sharp pointed drill, an opening is formed through to the point of the root, which, in some cases is very superficially situated. If there be any pus, it at once escapes, the congestion is relieved, and the inflammation subsides more or less rapidly — owing, of course, to the character of the patient — and the return of the disease is rendered less probable than can be realized from any other exclusive treatment; for, by this means, we get at the cause at once, which, having removed, we have accomplished that, without which, a speedy and permanent cure is rarely practicable. The treatment which I conceive to be next in importance to the former, and which I have employed in conjunction with it, is the administration of emetics and cathartics, whose virtues in the treatment of the diseases before us, consist in their depletory and derivative effects. The latter may be employed alone to good advantage in the forming stages of periostitis, as stated before, but not after suppuration has begun and proceeded to any considerable extent. Again, the most formidable cases of abscess with which we have to do, are those which affect often the inferior molars, and have proceeded so far as to destroy a great portion of the dental periosteum, and have become deep-seated in the soft tissues; the treatment the most proper here to be employed is immediate extraction, and compression externally. Having accomplished a cure without the sacrifice of the organ, the next step, in order to make the cure lasting, is to institute such means as are of a preventive nature, such as the exclusion of all foreign matter from the interior of the tooth, by filling with gold, the crown and root of the tooth. My practice is, where the case is the most favorable, to fill in the above manner. If not so favorable, I fill the crown only, and then form an opening through the gum about the eighth of an inch from its margin, in to the interior of the root; this admits of the escape of any matter that may accumulate in the tooth, where the healing process is sluggish.

